# Travel-Associated Dengue Cases — United States, 2010–2021

**DOI:** 10.15585/mmwr.mm7230a3

**Published:** 2023-07-28

**Authors:** Joshua M. Wong, Aidsa Rivera, Hannah R. Volkman, Brenda Torres-Velasquez, Dania M. Rodriguez, Gabriela Paz-Bailey, Laura E. Adams

**Affiliations:** 1 Division of Vector-Borne Diseases, National Center for Emerging and Zoonotic Infectious Diseases, CDC.

**Summary**

**What is already known about this topic?**

Dengue is the most common arboviral disease worldwide and a common cause of fever in travelers returning from areas with endemic disease. More dengue cases were reported to the World Health Organization in 2019 than in any other year. Vaccines are not currently recommended for travelers to areas with endemic dengue.

**What is added by this report?**

In 2019, the number of reported travel-associated dengue cases in the United States was 168% higher than the annual average during 2010–2018 and 2020–2021.

**What are the implications for public health practice?**

As dengue incidence increases globally, the risk for U.S. travelers will increase. Clinicians should be prepared to recognize, test for, and treat dengue. Travelers should follow CDC guidelines to prevent mosquito bites and vectorborne diseases.

## Abstract

Dengue, the leading cause of arboviral disease worldwide, can be fatal without appropriate treatment. Among 7,528 confirmed or probable travel-associated U.S. dengue cases reported during 2010–2021, one in five (1,474, 20%) was reported in 2019. This is 168% higher than the annual average number of cases reported during 2010–2018 and 2020–2021 (approximately 550 per year) and 61% higher than the 913 cases reported in 2016, the second highest year on record. The number of cases as a fraction of air traffic volume to international destinations outside North America or Europe was also highest in 2019, with 41.9 cases per million trips, compared with 21.0 per million in other years during 2010–2021. This report compares the number and characteristics of travel-associated dengue cases reported to national surveillance in the United States in 2019 with cases reported during 2010–2018 and 2020–2021. Areas with conditions suitable for dengue transmission as well as the population at risk for dengue are expected to increase, placing U.S. travelers at higher risk for infection. Health care providers should be aware that dengue is a common cause of fever in the returning traveler and be familiar with its signs and symptoms, testing, and management. Dengue vaccines are not currently recommended for U.S. travelers; therefore, persons should review areas of dengue risk and follow guidance for preventing mosquito bites.

## Introduction

Dengue is the leading cause of arboviral disease in the world (*1*) and can be fatal without appropriate treatment. In 2019, the World Health Organization (WHO) reported the highest number of dengue cases worldwide compared with cases reported in previous years (*2*).

Dengue is caused by four distinct but closely related dengue virus (DENV) types (1–4) and is transmitted by *Aedes* mosquitos. Infection with a DENV confers long-term immunity to that specific type but only short-term immunity to other types. Dengue causes approximately 400 million annual infections, one quarter of which lead to symptomatic disease, and results in more than 40,000 deaths.* In U.S. states, most dengue cases are associated with travel to areas with endemic dengue transmission (*3*), although endemic transmission does occur in six U.S. territories^†^ and freely associated states^§^ (*4*).

## Methods

Dengue has been a nationally notifiable disease since 2010. State health departments report dengue cases to CDC through CDC’s National Arbovirus Surveillance System (ArboNET), which maintains data on human disease and arboviral infections among presumptively viremic blood donors, veterinary disease cases, mosquitoes, dead birds, and sentinel animals.^¶^ This report includes confirmed and probable cases reported to ArboNET and associated with travel outside of the reporting jurisdiction within the 2 weeks preceding the onset of an acute febrile illness.

Confirmed or probable cases must have appropriate testing** and a clinically compatible case of dengue-like illness, dengue, or severe dengue.^††^ Cases per million air trips^§§^ by region of travel^¶¶^ were calculated using data on international air travelers from the National Travel and Tourism Office, as has been previously described*** (*3*). This activity was reviewed by CDC and was conducted consistent with applicable federal law and CDC policy.^†††^

## Results

**FIGURE 1:**
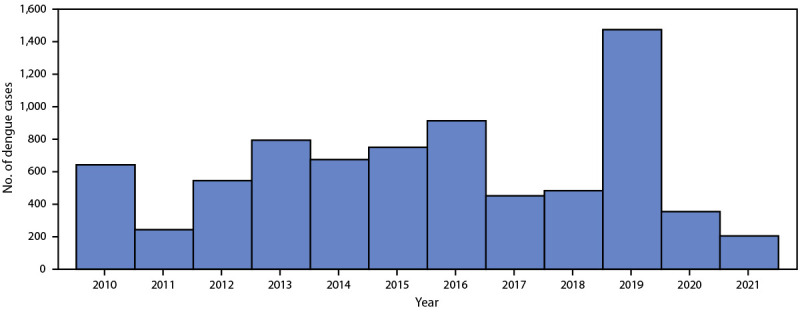
Reported confirmed and probable travel-associated dengue cases, by year (N = 7,528) — National Arbovirus Surveillance System, United States, 2010–2021

During 2010–2021, a total of 7,528 confirmed or probable travel-associated dengue cases were reported to ArboNET. Among these, 1,474 (20%) occurred in 2019, representing a 168% increase over the annual average of 550 cases during 2010–2018 and 2020–2021, and a 61% increase over the 913 cases reported in 2016, the year with the second highest number of cases (Figure 1). The lowest annual number of cases reported (205) was in 2021, when travel patterns were substantially altered because of the COVID-19 pandemic. During all three analysis periods, cases were evenly distributed among females and males, and age distribution remained consistent, with median ages of 41, 42, and 42 years during 2010–2018, 2019, and 2020–2022, respectively (Table).

**TABLE:** Characteristics of reported confirmed and probable travel-associated dengue cases (N = 7,528) — National Arbovirus Surveillance System, United States, 2010–2018, 2019, and 2020–2021

Characteristic	No. (%)		
	2010–2018	2019	2020–2021
**Total**	**5,495 (100)**	**1,474 (100)**	**559 (100)**
Case status*			
Probable	1,708 (31)	793 (46)	229 (41)
Confirmed	3,787 (69)	681 (54)	330 (59)
**Sex**			
Female	2,748 (50)	743 (50)	275 (49)
Male	2,746 (50)	729 (49)	284 (51)
Unknown	1 (<1)	2 (<1)	0 (—)
**Age group, yrs**			
Median (IQR)	41 (27–55)	42 (26–56)	42 (28–57)
0–9	143 (3)	68 (5)	21 (4)
10–19	557 (10)	178 (12)	60 (11)
20–29	962 (17)	196 (13)	80 (14)
30–39	936 (17)	238 (16)	88 (16)
40–49	991 (18)	254 (17)	107 (19)
50–59	986 (18)	256 (18)	94 (17)
≥60	910 (17)	284 (19)	108 (19)
Unknown	10 (<1)	0 (—)	1 (<1)
**Race^†^**			
American Indian or Alaska Native	17 (<1)	2 (<1)	3 (<1)
Asian or Pacific Islander	843 (15)	216 (15)	71 (13)
Black or African American	301 (6)	77 (5)	24 (4)
White	2,439 (44)	646 (44)	247 (44)
Other or unknown	1,895 (34)	533 (36)	214 (38)
**Ethnicity^†^**			
Hispanic or Latino	1,616 (30)	599 (41)	245 (44)
Non-Hispanic	2,538 (46)	504 (34)	189 (34)
Unknown	1,341 (24)	371 (25)	125 (22)
**Dengue clinical syndrome** ^§^			
Dengue-like illness	297 (5)	56 (4)	18 (3)
Dengue	5,030 (92)	1,388 (94)	532 (95)
Severe dengue	55 (1)	29 (2)	4 (1)
Unknown	113 (2)	1 (<1)	5 (1)
**Hospitalized**			
No	2,912 (53)	820 (56)	251 (45)
Yes	2,325 (42)	625 (42)	185 (33)
Unknown	258 (5)	29 (2)	123 (22)
**Outcome**			
Survived	5,310 (97)	1,405 (95)	521 (93)
Died	18 (<1)	1 (<1)	0 (—)
Unknown	167 (3)	68 (5)	38 (7)
**DENV type**			
DENV-1	131 (2)	51 (3)	43 (8)
DENV-2	79 (1)	32 (2)	35 (6)
DENV-3	51 (1)	23 (2)	16 (3)
DENV-4	29 (1)	1 (<1)	3 (<1)
Unknown	5,205 (95)	1,367 (93)	462 (83)
**Origin of acquisition**			
Outside of U.S. states or territories	4,830 (88)	1,414 (96)	497 (89)
Within a U.S. state or territory	443 (8)	13 (1)	25 (4)
Unknown	222 (4)	47 (3)	37 (7)
**Region of acquisition**			
Africa	97 (2)	18 (1)	12 (2)
Asia	1,615 (29)	401 (27)	107 (19)
Caribbean	1,794 (33)	570 (39)	149 (27)
Central America	684 (12)	158 (11)	34 (6)
Europe	5 (<1)	0 (—)	1 (<1)
North America^¶^	520 (10)	206 (14)	165 (30)
Oceania	127 (2)	35 (2)	13 (2)
South America	341 (6)	33 (2)	41 (7)
Multiple	90 (2)	6 (<1)	0 (—)
Unknown	222 (4)	47 (3)	37 (7)

**Abbreviations:** CSTE = Council of State and Territorial Epidemiologists; DENV = dengue virus; IgM = immunoglobulin M; NS1 = nonstructural protein 1.

* Case classification was performed according to the CSTE 2015 case definition (https://ndc.services.cdc.gov/case-definitions/dengue-virus-infections-2015/). Confirmed or probable cases must have a clinically compatible case of dengue-like illness, dengue, or severe dengue. The laboratory criterion for a probable case is defined as detection of IgM anti-DENV antibody in serum, if the person lived in or traveled to an area with transmission of another flavivirus or was recently vaccinated against a flavivirus (e.g., yellow fever virus or Japanese encephalitis virus). The laboratory criteria for a confirmed case are 1) DENV nucleic acid by reverse transcription–polymerase chain reaction in any body fluid or tissue, 2) DENV antigen in tissue by a validated assay, 3) DENV NS1 antigen by a validated assay, or 4) IgM anti-DENV antibody if exposure occurred in an area without evidence of other flavivirus transmission.

^†^ Persons of Hispanic or Latino (Hispanic) origin might be of any race but are categorized as Hispanic; all racial groups are non-Hispanic.

^§^ Clinical syndrome classification was updated in the CSTE 2015 case definition. Reported cases previously classified as “dengue hemorrhagic fever” and “dengue shock” before reporting changed were reclassified as “severe dengue,” and cases classified as “dengue fever with hemorrhage” were reclassified as “dengue” for this analysis. Annual trends might not be comparable.

^¶^ Travel-associated cases from North America include cases associated with travel to Mexico (882) and U.S. states (nine). Among cases associated with travel to U.S. states, four were reported during 2010–2018, three in 2019, and two during 2020–2021.

The proportions of cases classified as dengue-like illness, dengue, and severe dengue were similar in 2019, 2010–2018, and 2020–2021. The proportions of cases among patients who were hospitalized and who had an unknown disposition were similar during 2010–2018 (42% and 2%, respectively) and in 2019 (42% and 5%, respectively), whereas a smaller proportion of patients was hospitalized (33%) and a higher proportion had an unknown disposition (22%) during 2020–2021. Fewer than 1% of dengue patients died during 2010–2018 (18) and in 2019 (one), and no deaths occurred during 2020–2021. DENV-1 was the most frequently reported type across the three periods; however, the dengue type was unknown for 95% of cases during 2010–2018, 93% in 2019, and 83% during 2020–2021.

**FIGURE 2:**
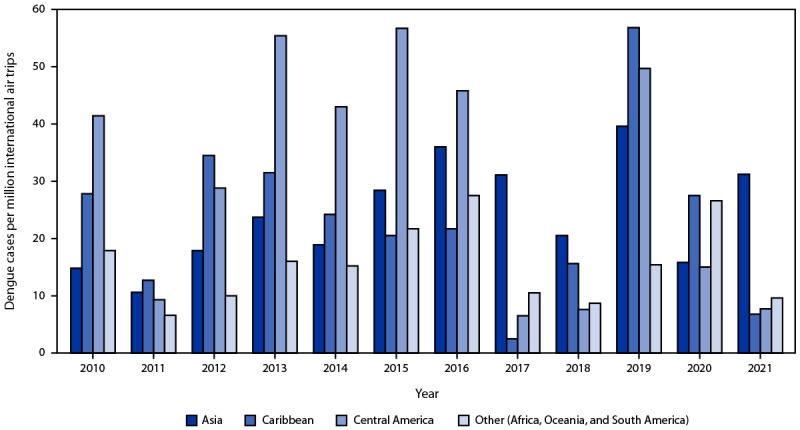
Reported confirmed and probable travel-associated dengue cases (N = 5,757)* per million international air trips, by region of acquisition — multiple data sources,^^†^^ United States, 2010–2021 **Abbreviations:** APIS = Customs and Border Protections Advance Passenger Information System; ArboNET = National Arbovirus Surveillance System. * The following cases were excluded: 1) those associated with travel to U.S. states or territories (481), because data from APIS excludes domestic travel; 2) those associated with travel to multiple regions (96) or where the country was unknown (306); 3) those associated with travel to North America (882), because travel between the continental United States and other North American countries commonly occurs by land borders, and the mode of travel for travel-associated cases is not reported to ArboNET; and 4) those associated with travel to Europe (six). ^^†^^ International air traffic volume information (denominator) is from the APIS/I-92 Monitor (https://www.trade.gov/us-international-air-travel-statistics-i-92-data) and from the Survey of International Air Travelers (https://www.trade.gov/survey-international-air-travelers-siat), both managed by the U.S. Department of Commerce, National Travel and Tourism Office.

During the entire period, most cases (90%) were associated with travel outside U.S. states or territories. The most frequently visited region among travel-associated cases in 2019 was the Caribbean (39%), followed by Asia (27%) and North America^§§§^ (14%). Travel patterns were similar during 2010–2018, with 33%, 29%, and 10% of patients reporting travel to those three regions, respectively. However, during 2020–2021, a period with major disruptions to travel because of the COVID-19 pandemic, the most frequently visited region was North America (30%), followed by the Caribbean (27%) and Asia (19%). The number of dengue cases per million air trips to destinations outside North America or Europe in 2019 (41.9 per million) was nearly twice that during other years during 2010–2021 (21.0 per million). These rates varied by region of travel across the periods analyzed. During 2010–2018, the highest number of cases per million air trips was associated with travel to Central America (32.1), followed by Asia (22.9) and the Caribbean (20.5). (Figure 2) (Supplementary Table 1, https://stacks.cdc.gov/view/cdc/131003). In 2019, the highest rates were associated with travel to the Caribbean (56.8), Central America (49.7), and Asia (39.6); during 2020–2021, the highest number of cases per million trips (37.3) was associated with travel to Oceania, followed by Asia (23.5) and South America (15.8).

 Travelers returning from the top 10 countries of acquisition during 2010–2021 accounted for more than two thirds (69%) of cases reporting an international travel history (Supplementary Figure; https://stacks.cdc.gov/view/cdc/131002). Seven countries (Cuba, the Dominican Republic, El Salvador, India, Jamaica, Mexico, and the Philippines) were among the top 10 countries of acquisition across all three periods (Supplementary Table 2, https://stacks.cdc.gov/view/cdc/131004). Seasonality among travel-associated cases was similar during 2010–2018 and 2019, with most cases occurring during July–November. Seasonal trends were less apparent during 2020–2021, when fewer cases were reported relative to previous periods.

## Discussion

U.S. jurisdictions reported more travel-associated dengue cases in 2019 than in any other year since dengue in the United States became nationally notifiable in ArboNET in 2010. The lowest number of cases reported occurred in 2021, during a period marked by unprecedented travel restrictions and a decline in overall travel because of the COVID-19 pandemic. The characteristics of persons with dengue, including age, sex, clinical syndromes, and outcomes, were similar in 2019 to those reported in other years during 2010–2021. The Caribbean, Asia, and North America were the top regions of acquisition, respectively, during 2010–2018 and 2019. However, during 2020–2021, the proportion of cases associated with travel to North America surpassed both the Caribbean and Asia, and the proportion of cases associated with travel to Asia decreased relative to the Caribbean, possibly reflecting a decline in overall travel because of the COVID-19 pandemic, with the largest regional decrease in air trips to Asia, or variations in regional dengue activity during those periods. The number of cases per million international air trips was higher in 2019 than that during 2010–2018 or 2020–2021, which varied by year and region of travel. The sharp overall increase in 2019 mirrors global dengue activity, with the highest number of dengue cases worldwide reported to WHO in 2019 (*2*) and in the Region of the Americas (*5*) since reporting to the Pan American Health Organization/WHO began in 1980.^¶¶¶^

### Limitations

The findings in this report are subject to at least four limitations. First, case counts are underestimated because many travelers with dengue do not seek medical care, are not tested for dengue when evaluated, or do not receive a correct diagnosis. Second, incomplete reporting of clinical data might lead to misclassification of the clinical syndrome, likely underestimating severe dengue cases among reported cases. Third, changes to the clinical syndrome classification in the 2015 case definition required reclassification of cases reported before this change for this analysis, complicating its comparison among the three periods. Finally, the dataset does not include cases among U.S. travelers who contracted dengue while traveling and whose cases were not reported to U.S. surveillance.

### Implications for Public Health Practice

Global dengue is expected to increase in disease prevalence and geographic range, placing U.S. travelers at increased risk for infection (*1*). As travel returns to levels similar to those before the COVID-19 pandemic, clinicians should consider dengue in the differential diagnosis of fever in the returning traveler and understand its signs and symptoms, appropriate testing, and disease management**** for two reasons: 1) early recognition of dengue and prompt intravenous fluid management, when indicated, reduces mortality to <1%, whereas untreated dengue can have a case-fatality ratio as high as 13% (*1*) and 2) travelers infected with dengue returning to the United States can introduce the virus to local *Aedes* mosquito populations, present in one half of U.S. counties (*6*), potentially leading to local DENV transmission.^††††^ It is important for jurisdictions to strengthen surveillance for dengue and consider ways to increase the identification and reporting of type.^§§§§^ Because persons build immunity against each specific DENV type, surveillance that can reliably detect the introduction of new types will guide epidemic risk potential and the impact of vaccine and vector control interventions in areas where dengue is endemic.

Although a dengue vaccine is recommended for routine use in children and adolescents aged 9–16 years with laboratory-confirmed previous DENV infection who live in areas of the United States where dengue is endemic (*7*,*8*), vaccination is not recommended for travelers.^¶¶¶¶^ Dengue and other vectorborne diseases such as Zika and malaria***** can be prevented while traveling by taking measures to prevent mosquito bites,^†††††^ including using Environmental Protection Agency–registered insect repellent, wearing protective clothing,^§§§§§^ and staying in lodging that has air conditioning or window screens. New interventions are emerging, including new dengue vaccines in clinical trials and novel vector control methods that do not rely on chemical control of mosquitoes (*1*,*9*). Effective and scalable public health measures to prevent dengue will be needed to reduce risk among residents of and travelers to areas where dengue is endemic.

## Footnotes

*
https://www.cdc.gov/dengue/about/index.html

† American Samoa, Puerto Rico, and U.S. Virgin Islands.

§ Federated States of Micronesia, Marshall Islands, and Palau.

¶
https://www.cdc.gov/mosquitoes/mosquito-control/professionals/ArboNET.html

** Case classification was performed according to the Council of State and Territorial Epidemiologists’ (CSTE) 2015 case definition (https://ndc.services.cdc.gov/case-definitions/dengue-virus-infections-2015/). Confirmed or probable cases must have a clinically compatible case of dengue-like illness, dengue, or severe dengue. The laboratory criterion for a probable case is defined as detection of immunoglobulin M (IgM) anti-DENV antibody in serum, if the person lived in or traveled to an area with transmission of another flavivirus or was recently vaccinated against a flavivirus (e.g., yellow fever virus or Japanese encephalitis virus). The laboratory criteria for a confirmed case are detection of 1) DENV nucleic acid by reverse transcription–polymerase chain reaction in any body fluid or tissue, 2) DENV antigen in tissue by a validated assay, 3) DENV nonstructural protein 1 (NS1) antigen by a validated assay, or 4) IgM anti-DENV antibody if exposure occurred in an area without evidence of other flavivirus transmission.

†† Clinical syndrome classification was updated in the 2015 CSTE case definition. Reported cases previously classified as “dengue hemorrhagic fever” and “dengue shock” before reporting changed were reclassified as “severe dengue,” and cases classified as “dengue fever with hemorrhage” were reclassified as “dengue” for this analysis. Annual trends might not be comparable.

§§ Individual travelers might take multiple international air trips every year, each of which is counted separately in the total number of air trips. Because data on total number of international air travelers and their region of travel are not available, true incidence (cases per number of international air travelers per year) cannot be calculated.

¶¶ The following cases were excluded: 1) those associated with travel to U.S. states or territories (481), because data from the Customs and Border Protection Advance Passenger Information System (APIS) excludes domestic travel; 2) those associated with travel to multiple regions (96) or where the country was unknown (306); 3) those associated with travel to North America (882), because travel between the continental United States and other North American countries commonly occurs by land borders, and the mode of travel for travel-associated cases is not reported to ArboNET; and 4) those associated with travel to Europe (six).

*** International air traffic volume information (denominator) is from the APIS/I-92 Monitor (https://www.trade.gov/us-international-air-travel-statistics-i-92-data) and from the Survey of International Air Travelers (https://www.trade.gov/survey-international-air-travelers-siat), both managed by the U.S. Department of Commerce, National Travel and Tourism Office.

††† 45 C.F.R. part 46.102(l)(2), 21 C.F.R. part 56; 42 U.S.C. Sect. 241(d); 5 U.S.C. Sect. 552a; 44 U.S.C. Sect. 3501 et seq.

§§§ Travel-associated cases from North America include cases associated with travel to Mexico (882) and the United States (nine). Among cases associated with travel to the United States, four were reported during 2010–2018, three in 2019, and two during 2020–2021.

¶¶¶
https://www3.paho.org/data/index.php/en/mnu-topics/indicadores-dengue-en.html

****
https://www.cdc.gov/dengue/healthcare-providers/index.html

†††† Local dengue outbreaks were recently reported in Texas in 2013, Hawaii in 2015, and Florida in 2013 and 2020. https://www.cdc.gov/dengue/areaswithrisk/in-the-us.html

§§§§ CDC Dengue Branch provides free dengue testing to public health laboratories requesting confirmatory testing and virus typing (https://www.cdc.gov/ncezid/dvbd/specimensub/dengue-shipping.html). Reporting DENV type is preferred, but not required. https://ndc.services.cdc.gov/mmgpage/arboviral-message-mapping-guide/

¶¶¶¶
https://www.cdc.gov/dengue/vaccine/index.html

*****
https://www.cdc.gov/malaria/travelers/index.html

†††††
https://www.cdc.gov/dengue/prevention/plan-for-travel.html

§§§§§ Protective clothing includes loose-fitting, long-sleeved shirts and pants.
